# Nanoscale Analysis of Sulfur Poisoning Effects on Hydrogen Sorption in Single Pd Nanoparticles

**DOI:** 10.1021/acsnano.5c08917

**Published:** 2025-10-15

**Authors:** Mazal Kostan-Carmiel, Athanasios Theodoridis, Helen R. Eisenberg, Tamar Stein, Christoph Langhammer, Elad Gross

**Affiliations:** † Institute of Chemistry, 1438 The Hebrew University, Jerusalem 9190401, Israel; ‡ Center for Nanoscience and Nanotechnology, The Hebrew University, Jerusalem 9190401, Israel; § Department of Physics, 11248 Chalmers University of Technology, Gothenburg SE-412 96, Sweden; ∥ Fritz Haber Research Center for Molecular Dynamics, The Hebrew University, Jerusalem 9190401, Israel

**Keywords:** hydrogen sensors, palladium, sulfur poisoning, nanospectroscopy, nanoparticles

## Abstract

Hydrogen gas is rapidly approaching a global breakthrough as a carbon-free energy source. In such a hydrogen economy, safety sensors for hydrogen leak monitoring will be an indispensable element due to the high flammability of hydrogen–air mixtures. Palladium-based nanoparticles function as optical hydrogen sensors due to their ability to reversibly absorb hydrogen and undergo a phase transition to palladium hydride, which induces a spectral shift in their localized plasmon resonance. However, the effectiveness of palladium-based nanoparticles as hydrogen sensors is compromised in realistic environments due to surface poisoning from various contaminants, including sulfur-containing compounds (SO_*x*_), which block active sites required for hydrogen dissociation. In this study, we use atomic force microscopy, infrared nanospectroscopy, and Kelvin probe force microscopy, in addition to density functional theory (DFT) calculations, to investigate the impact of SO_*x*_ poisoning on the hydrogen sorption dynamics of single Pd nanoparticles. It is demonstrated that SO_*x*_ preferentially adsorbs on the particle’s rim, significantly altering the kinetics of hydrogen (de)­sorption and lowering the total sorption capacity. Single particle analysis revealed that poisoning leads to slower (de)­sorption kinetics due to blocking of highly reactive surface sites that are located on the particle’s rim. DFT calculations show that SO_*x*_ binds significantly less strongly to the flat palladium hydride surface compared to the flat palladium surface and the rough surface found at the nanoparticle rim. These calculations rationalize the selective desorption of SO_*x*_ from the center of the nanoparticle following exposure to hydrogen and its persistent binding to the particle rim.

## Graphical abstract

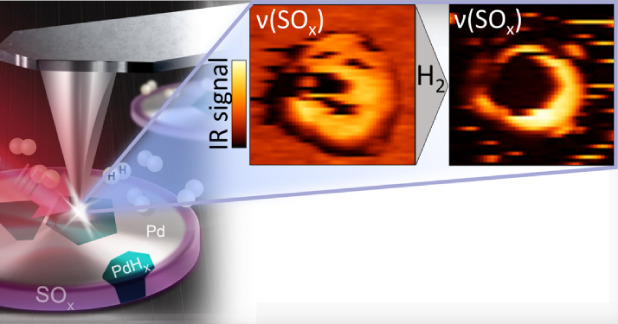

## Introduction

The use of hydrogen-based energy systems requires fast, robust, and cost-efficient safety sensors for leak detection, as well as for process monitoring in complex chemical environments.^1,2^ In this context, Pd-based and Pd-alloy-based nanoparticles (NPs) are very attractive as optical sensors for H_2_ detection, and pure Pd is widely used as a prototype system for their development^3–7^ since H_2_ molecules exhibit barrier-less dissociation on Pd surfaces. Once dissociated, hydrogen atoms (H) can occupy interstitial lattice sites to form a solid solution at low hydrogen concentration and, above a critical concentration, undergo first-order phase transformation into a Pd-hydride phase. The formation of a solid solution or a Pd-hydride modifies both the volume and electronic properties of Pd in general, and of Pd NPs in particular.^8–13^ Specifically, the changes in the electronic properties associated with the transition from the metallic to hydride phase result in a distinct spectral shift of the localized surface plasmon resonance (LSPR) of the nanoparticles, allowing sensitive optical detection of hydrogen.^5,6,13–17^ The hydride phase can be reversed into the metallic state by lowering the H_2_ partial pressure, making the phase transition fully reversible, thus allowing reversible switching of the plasmonic resonances of Pd nanostructures for multicycle hydrogen sensors.^18,19^

One of the main challenges in using Pd NPs, as well as Pd-based alloys, as hydrogen sensors in realistic environments, such as ambient air, is their high susceptibility to contamination and deactivation by species like H_2_O, CO, NO_*x*_ and SO_*x*_.^20–24^ Sulfur-containing species, such as SO_*x*_, are specifically problematic since they can bind to Pd NPs in a strong and often irreversible way, resulting in catalytic deactivation due to the blocking of surface sites that are required for H_2_ dissociation.^25,26^ Thus, sulfur is one of the most effective poisoners of Pd surfaces, and analysis of its impact on the mechanism of hydrogen (de)­sorption in/from Pd NPs can provide understanding about the role of poisoners in general and of sulfur in particular. Such a mechanistic understanding is essential, for example, for the design of Pd-based hydrogen sensors with a shorter response time and high deactivation resistance in realistic application environments.

In this context, due to both inter- and intraparticle heterogeneity in active site distribution,^27–32^ it is essential to use experimental methods that will provide nanometer-scale spatial resolution and nanoscale chemical analysis,^33–40^ to probe the distribution of poisoners on the NP surface and their impact on hydrogen (de)­sorption. To this end, significant progress in nanoscale analysis of the diffusion of hydrogen in Pd NPs, and of the distribution of SO_*x*_ on Pd NPs, has recently been reported using high spatial resolution microscopy and spectroscopy.^41–43^ Specifically, IR nanospectroscopy measurements revealed that the types of SO_*x*_ adsorbates and their adsorption configurations show significant heterogeneity due to dissimilarities in the surface morphologies of individual Pd NPs. It was also identified that the hydrogenation process, in the absence of poisoners, is highly influenced by the nanoscale morphology and that hydrogen sorption is not necessarily facilitated throughout the entire nanoparticle.^44–51^ However, no reports address the interplay of SO_*x*_ poisoning and hydrogen sorption in Pd NPs despite their critical importance for generating a fundamental understanding of the detrimental impact of such poisoners on the performance of Pd-based hydrogen sensors.

In this work, we employed *operando* Atomic Force Microscopy (AFM), IR nanospectroscopy, and Kelvin-probe force microscopy (KPFM) measurements to monitor the nanoscopic influence of sulfur poisoning on hydrogen (de)­sorption of/from single Pd NPs (Figure 1). It was recently demonstrated by us and others that tip-based high-resolution nanospectroscopy measurements can effectively map the reactivity pattern of catalytic materials.^52–65^ Herein, we analyzed the distribution of the SO_*x*_ species on the surface of Pd NPs by IR nanospectroscopy measurements.

**Figure 1:**
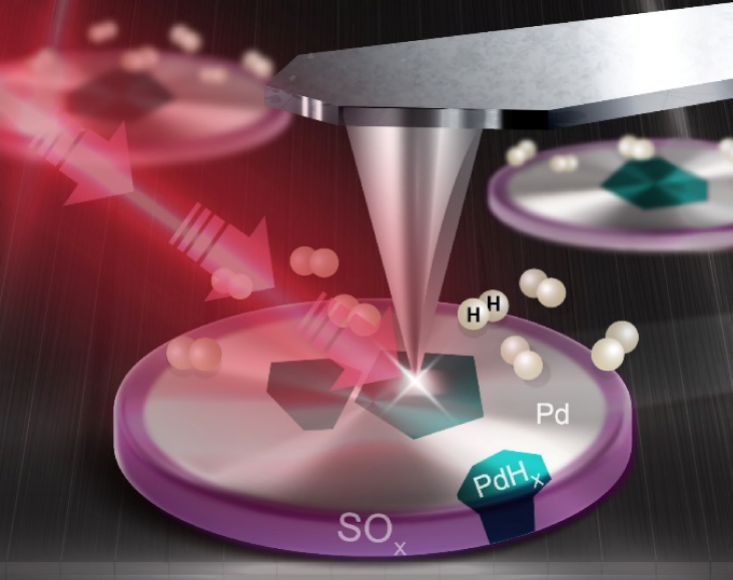
Schematic representation of the experimental setup. Disk-shaped Pd NPs were nanofabricated on an oxidized Si wafer using hole-mask colloidal lithography.^66^ Subsequently, they were exposed to H_2_SO_4_ to simulate sulfur poisoning and then exposed to alternating cycles of H_2_ and N_2_. IR nanospectroscopy was employed to map the spatial distribution of SO_*x*_ species on the surface of randomly selected single Pd NPs. AFM topography measurements probed reversible changes in the particle’s volume that were induced by hydrogen (de)­sorption, while KPFM measurements monitored the corresponding reversible changes in the electronic properties, transitioning between metallic Pd and Pd hydride.

Topography measurements of pristine and poisoned Pd NPs were performed under alternating N_2_ and H_2_ environments to probe the changes in NPs’ topography, induced by hydrogen (de)­sorption. Finally, we used KPFM measurements to monitor the electronic changes in the NPs following their transformation from metallic Pd to Pd hydride. These measurements, along with *ab initio* Density Functional Theory (DFT) calculations, allowed us to unravel the connection between the spatial distribution of SO_*x*_ species on single Pd NPs and their impact on hydrogen (de)­sorption and phase changes in these NPs. We identified that exposure of sulfur-poisoned particles to H_2_ induced a selective removal of sulfur from the particles’ surface while leaving residues on highly reactive surface sites, mostly located at the particles’ rim. Sulfur poisoning on the particle’s periphery led to a decrease in the hydrogen (de)­sorption kinetic rate and reduced the overall hydrogen uptake.

## Results and Discussion

Pd NPs, with a diameter of 190 ± 10 nm and a height of 25 ± 5 nm (Figure S1), were prepared on a Si (100) wafer with a native oxide layer by using hole-mask Colloidal Lithography nanofabrication (see the Supporting Information for additional details).^66^ Subsequently, the sample was immersed in H_2_SO_4_ (10 mM, 25 °C, 10 min), followed by heating the dried sample in air (110 °C, 10 min) to remove physisorbed residues.^43^ Following this process, X-ray photoelectron spectroscopy (XPS) measurements were conducted and revealed a 0.7 ± 0.1% atomic percentage of sulfur on the sample.

AFM and AFM-IR measurements were performed to analyze the influence of H_2_SO_4_ exposure on the topography of the NPs and to reveal the distribution of SO_*x*_ species on their surface (Figure 2). AFM measurements were conducted before and after the exposure of the Pd NPs to H_2_SO_4_ (Figure 2a,c, respectively). Height profile and corrugation analyses were performed on particles exposed to H_2_SO_4_, revealing an increase of up to 1.5 ± 0.2 nm (∼6%) in height and a 3.8% increase in surface roughness, as analyzed by AFM measurements. These structural changes are attributed to the partial dissociation of H_2_SO_4_ on the Pd surface and subsequent hydrogen sorption, as discussed further.

**Figure 2:**
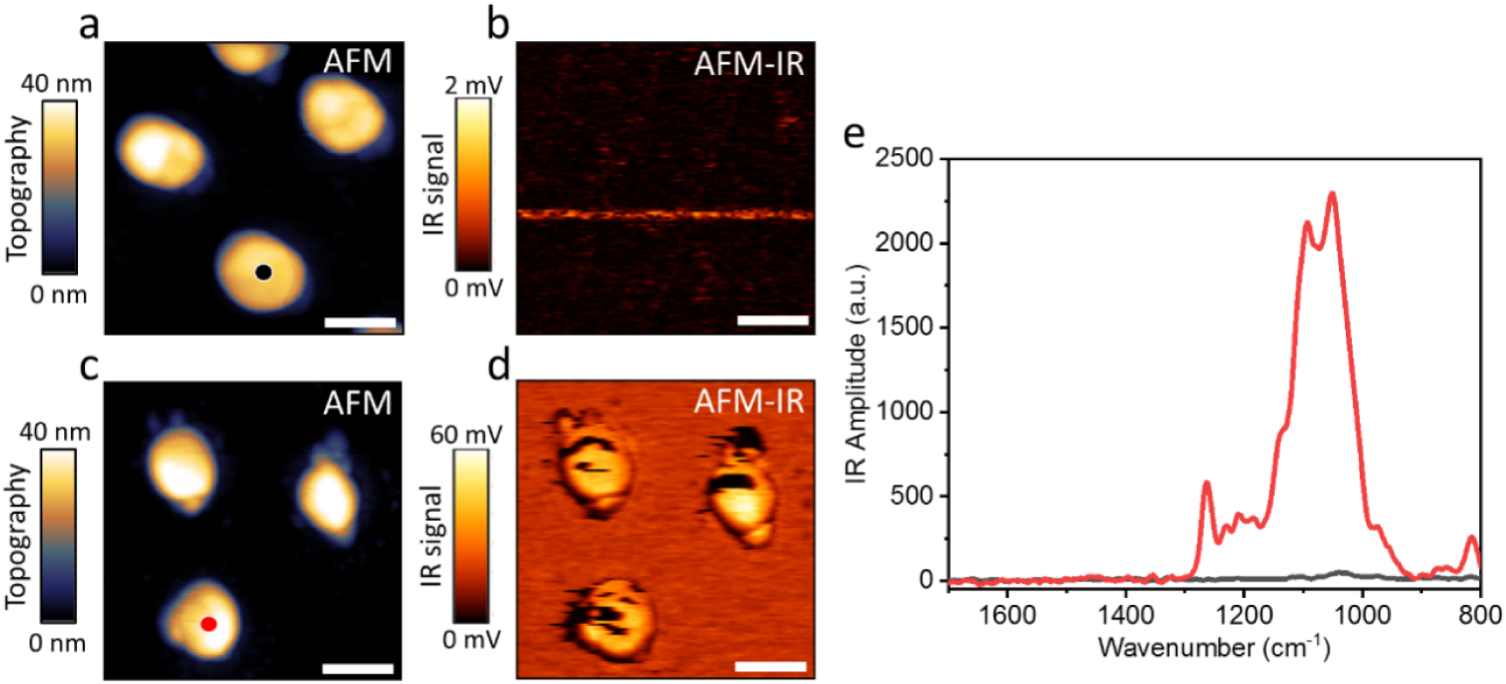
AFM topography images and AFM-IR mapping at 1108 cm^–1^ of pristine Pd NPs (a and b, respectively) and after their exposure to H_2_SO_4_ (c and d, respectively). (e) IR spectra were acquired from the center of a Pd NP before (black spectrum) and after (red spectrum) exposure to H_2_SO_4_. The IR measurement locations are indicated by black and red circles in (a) and (c), respectively. The scale bar represents 200 nm.

AFM-IR mapping at 1108 cm^–1^, correlated to SO_*x*_ species,^43^ showed no signal prior to the exposure of the NPs to H_2_SO_4_ (Figure 2b), while a distinctive signal was identified after exposure to H_2_SO_4_ (Figure 2d). This demonstrates that the Pd NPs were coated with SO_*x*_, along with a weaker vibrational signature on the SiO_2_/Si substrate. A coating coverage of 56 ± 2% was identified on Pd NPs by the analysis of the AFM-IR images. The incomplete coverage is mostly attributed to local glitches in the AFM-IR mapping, as evidenced by vertical lines on the particle surface, where no IR signal was detected. It is hypothesized that these glitches arise from local variations in particle height, which limit the ability of the tip to properly track the IR signal across these sites.

An IR spectrum was acquired from the center of a single Pd NP, prior to exposure to H_2_SO_4_, and showed a low signal at 1035 cm^–1^ (Figure 2e, black-colored spectrum). This signal can be associated with Si–O stretches from the silicon substrate, which can be probed through the thin Pd NP.^67^ After exposure to H_2_SO_4_, a much more distinct IR signal was detected (Figure 2e, red-colored spectrum) with peaks at 1108 and 1190 cm^–1^, which are attributed to SO_*x*_ adsorbed on metallic Pd^0^ and Pd^*x*+^, respectively, as recently identified based on DFT calculations.^43^ The presence of an oxidized Pd species, following exposure to H_2_SO_4_, was also probed in XPS measurements (Figure S2). The peak at 1280 cm^–1^ is attributed to S=O vibration of surface-bound sulfate/bisulfate, while the peak at 810 cm^–1^ correlates to the bisulfate S–OH stretch due to interaction with water residues, and the shoulder at ∼1000 cm^– 1^ can also be assigned to the S–OH stretch.^68,69^

To subsequently monitor the influence of SO_*x*_ poisoning on hydrogen (de)­sorption into/from the Pd NPs, we measured changes in their topography during exposure to alternating H_2_ and N_2_ cycles. For this purpose, we positioned the sample in a gas flow cell (10 mL in volume) in which the IR signal was acquired *in situ*, with single particle resolution, as schematically illustrated in Figure 1. The sample was first exposed to 1 atm of N_2_ for 60 min and subsequently to 1 atm of a N_2_:H_2_ mixture with a 100:1 ratio for 60 min at a constant flow rate of 0.5 L/min. This mixing ratio was designed to be above the pressure threshold for the Pd to PdH_*x*_ phase transition and thus enabled monitoring the hydride formation and phase transition on single nanoparticles.^21^ The particles’ topography was analyzed once the sample reached a state in which no noticeable changes in the particle’s topography were recorded within the AFM acquisition duration.

Height profile analysis was performed for single Pd NPs following exposure to alternating gas environments (Figure 3a–c), revealing that the alternating gas environments led to reversible changes in the height and diameter of the NPs (Figure 3c). In order to quantify the changes that are induced by exposure to H_2_ and N_2_, the variations in the diameter were measured at four different points across the NPs, and the changes in the height were measured at five different points around the center of the NP (Figure 3d). Height and diameter differences were extracted by measuring the values at the same positions following consecutive exposures to H_2_ and N_2_ (Figure 3e,f). Repeated measurements were conducted to verify that the gas environment does not noticeably influence the accuracy of the AFM measurements.

**Figure 3:**
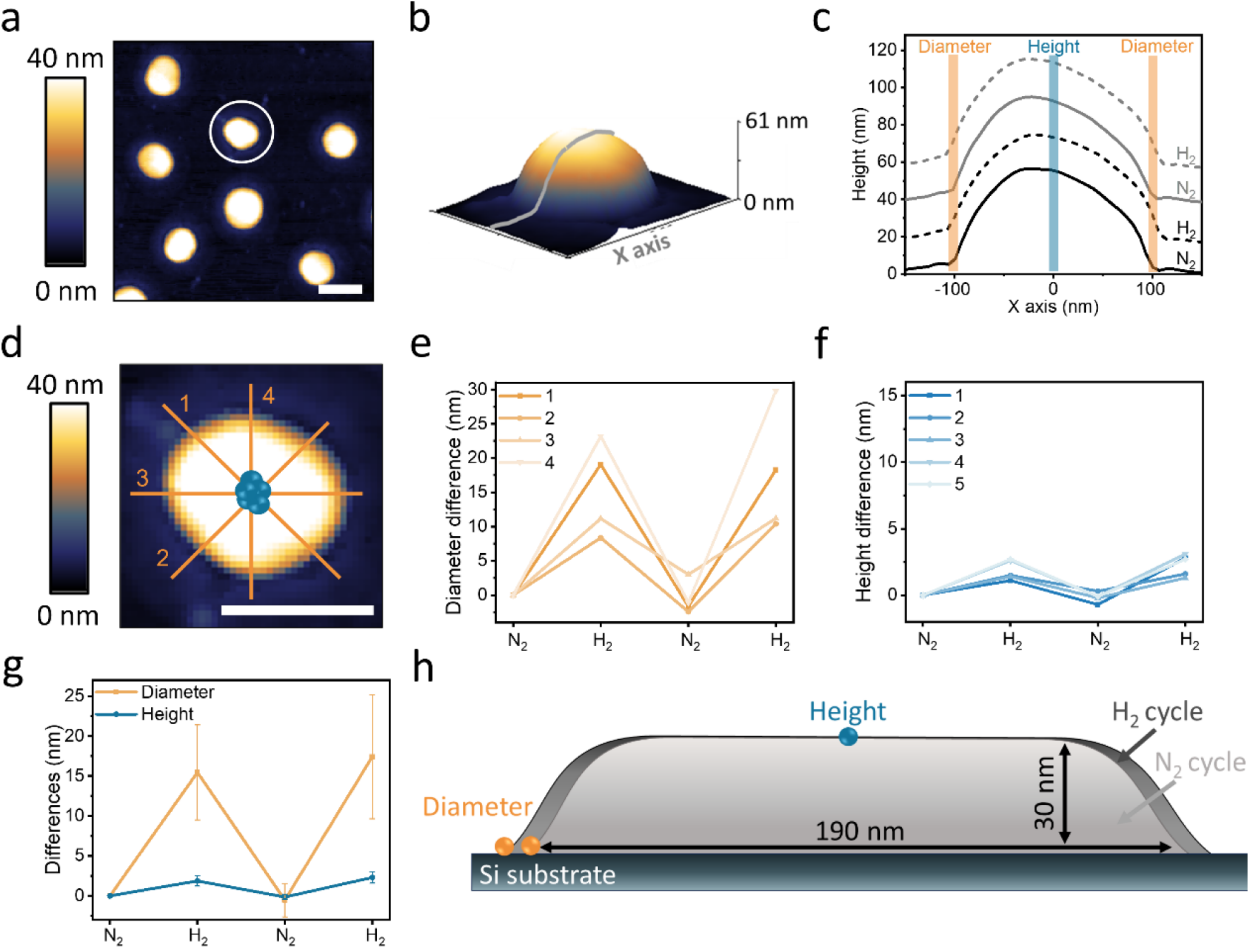
(a) AFM topography image of multiple Pd nanoparticles (NPs), with the white circle highlighting the particle analyzed in detail in the subsequent panels (scale bar: 200 nm). (b) 3D rendering of the Pd NP, illustrating a typical height profile measurement. (c) Height profiles measured along the axis of the particle shown in (d) after exposure to two consecutive cycles of N_2_ (solid lines) and H_2_ (dotted lines). The height profiles measured after the first and second cycles in (c) are colored in black and gray, respectively. The profiles are vertically offset by 20 nm along the *y*-axis for clarity. Diameter and height variations induced by alternating gas exposure are analyzed by measuring the diameter at four positions across the NP (yellow lines in d) and the height at five points around its central region (blue circles in d). The resulting diameter and height changes after exposure to N_2_ and H_2_ are presented in (e) and (f), respectively. (g) Averaged diameter and height changes, calculated from the data in (e) and (f). (h) Schematic (not to scale) illustration of the NP’s profile changes induced by exposure to N_2_ and H_2_.

Quantitative analysis of the changes in the particle’s dimensions, based on the measurements that were described above, shows that H_2_ sorption led to an increase of 9.2% (17.4 ± 7.7 nm) in the particle’s diameter (Figure 3g). The height in the central part of the particle increased by 7.7% (2.3 ± 0.7 nm) following exposure to H_2_. Larger intraparticle variations in diameter were obtained in comparison to height variations (as shown in Figure 3e,f, respectively) and are likely the consequence of significant structural heterogeneities along the rim due to, e.g., differently oriented crystallites within the particle.^70^

Similar morphological analysis was performed on five pristine Pd NPs and revealed a significant diversity in the influence of varying gas environments on changes in their height and diameter (Figures 4a–d and S3). While particle P2 (Figure 4a) exhibited highly reversible volume changes (as described in its detailed analysis in Figure 3 and in the blue-colored curves in Figure 4b,c), other particles showed less uniform volume change patterns. These variations are attributed to differences in nanoparticle morphology, which have an inherent effect on their hydrogen (de)­sorption affinity. The degree of hydrogen (de)­sorption affinity and its reversibility varies among different nanoparticles due to inter- and intraparticle structural heterogeneity. In particular, grain boundaries, twin planes, and other defects modify the hydrogen diffusion pathways.^70^

**Figure 4:**
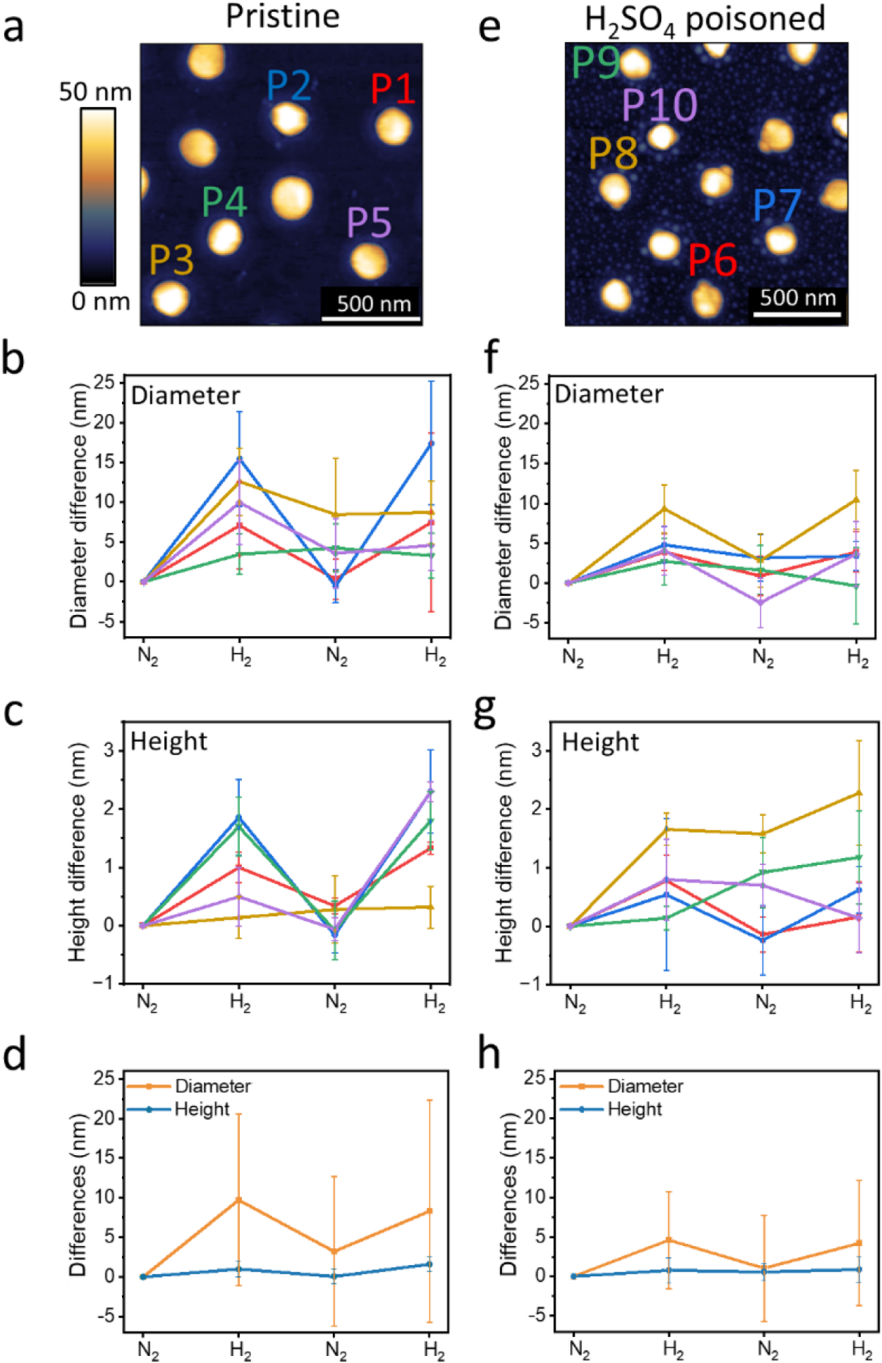
Topography and morphology analysis of pristine (a–d) and sulfur-poisoned (e–h) Pd NPs. AFM topography images of pristine and poisoned NPs are shown in (a) and (e), respectively. The AFM images of the pristine and poisoned NPs were acquired in different areas of the sample, and different NPs were measured on the pristine and poisoned samples. Average of the diameter and height variations of pristine NPs (b and c, respectively) and those of poisoned NPs (f and g, respectively) following exposure to N_2_ and H_2_ cycles. The color coding in (b) and (c) and (f) and (g) corresponds to the colors of the nanoparticles shown in (a) and (b), respectively. The average diameter and height changes of five pristine and five poisoned NPs are shown in (d) and (h), respectively.

Morphological variances, following exposure to alternating gas environments, were enhanced in the particle’s diameter compared to the particle’s height (Figure 4b,c, respectively). FIB-TEM analysis of several NPs uncovered structural heterogeneities in the NPs, revealing the presence of twin and high-angle grain boundaries in the NP (Figure S4). These structural variations between NPs can impact the sorption kinetics and volume expansion, e.g., due to enhanced hydrogen diffusion along grain boundaries and crystallite-orientation-dependent lattice strain. In the rim region, two NPs out of five showed a reversible increase and decrease in their height upon cycling, while three out of five NPs did not show the expected reversibility (Figure 4b). In the center part of the particles, however, four out of five particles exhibited reversible height changes (Figure 4c). On average, across all five analyzed particles, we recorded an increase of 4.4% (8.3 nm) in the diameter of the NP and a height increase of 5.3% (1.6 nm) in the central part of the particle (Figure 4d). These changes yield a volume increase of 11.3%, following exposure to H_2_ (Figure S5), which is in reasonable agreement with the 10.4% volume increase reported for bulk Pd.^71^

A different hydrogen (de)­sorption signature was identified for Pd NPs following their exposure to H_2_SO_4_ (Figures 4e–h and S6). Quantitative analysis shows that H_2_ sorption led to an average increase of 2.2% and 2.9% in diameter and height, respectively (Figure 4h), yielding a volume increase of 5.4% following exposure to H_2_ (Figure S5). Thus, particles’ poisoning lowered the diameter and height growth by 50% and 55%, respectively.

AFM-IR mapping was performed to identify the influence of H_2_ sorption on the spatial distribution of SO_*X*_ on the poisoned Pd NPs (Figure 5). A distinctive IR signal at 1108 cm^–1^ was detected on the NPs’ surface prior to exposure to hydrogen (Figure 5a). A 4-fold lower signal was detected on the bare silica surface between the nanoparticles. SO_*x*_ IR signals were reduced in intensity following exposure to the first and second cycles of H_2_ (Figure 5b,d, respectively), indicating SO_*x*_ desorption from both the NPs and substrate surfaces induced by hydrogen. It is hypothesized that desorption from the silica surface is facilitated by a reductive mechanism. Dissociative chemisorption followed by spillover of reactive hydrogen atoms from the Pd NPs can reduce adsorbed sulfate species, which are formed through interaction with surface silanol groups via hydrogen bonding or proton transfer, leading to the release of volatile products.^72,73^ Desorption of chemisorbed SO_x_ from the central part of the Pd NP is attributed to the formation of PdH, which lowers the binding energy of the chemisorbed SO_x_ species, as supported by DFT calculations, which will be discussed in the following paragraphs.

**Figure 5:**
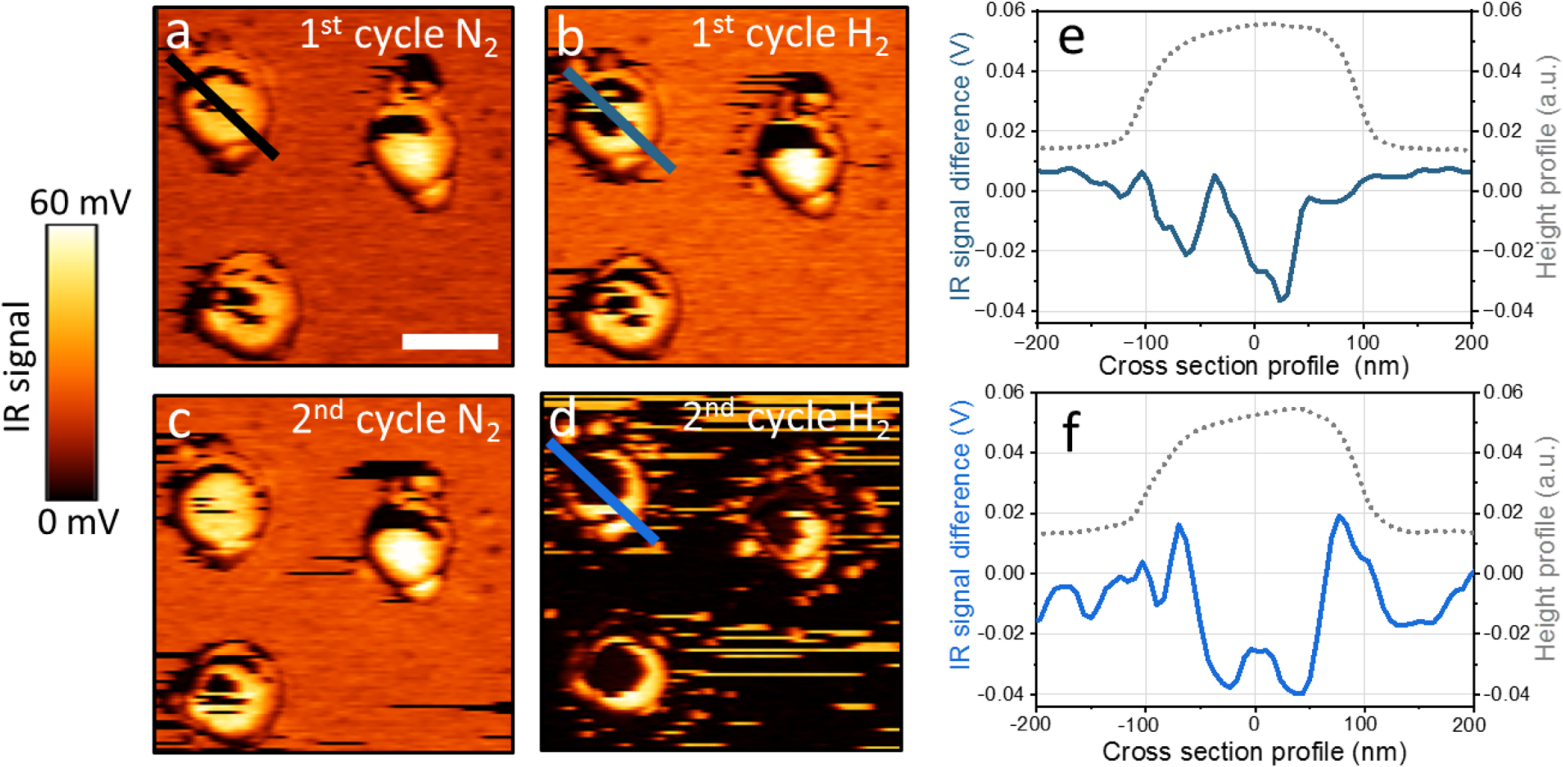
AFM-IR mapping of poisoned Pd NPs was acquired at 1108 cm^–1^ following exposure to N_2_ (a) and H_2_ (b) and a second cycle of N_2_ (c) and H_2_ (d). IR signal differences (IR signal­(H_2_) – IR signal­(N_2_)) were measured after the first and second H_2_ exposure cycles and are plotted (blue) in (e) and (f), respectively, along with the AFM height profile (black-colored). IR signal differences shown in (e) and (f) were measured across the lines marked in (a), (b), and (d). The scale bar represents 200 nm.

Analysis of the changes in the IR signal across the NPs showed a decrease in the signal amplitude of SO_*x*_ in the central part of the particle after the first cycle of H_2_ exposure, while the signal along the particle’s periphery did not change noticeably (Figure 5e). An increase in the IR signal was observed across the NP during the second cycle of N_2_ exposure, potentially attributed to SO_*x*_ adsorption following its diffusion from the silicon surface to the stronger binding sites on the Pd NPs.

The IR signal in the central part of the particle was further reduced following a second H_2_ exposure cycle, after which the SO_*x*_ signal was detected only along the rim of the NP (Figure 5f). An increase was observed in the IR signal at the rim of the NP (Figure 5f), which is attributed to the adsorption of SO_*x*_ species that diffused from the particle center or from the silica surface and subsequently accumulated at the rim. The energetic justification for the diffusion of SO_*x*_ from the central part of the particle to its rim is rationalized by DFT calculations that will be discussed in the following paragraphs. Analysis of the IR signal across several NPs demonstrated the selective removal of SO_*x*_ (Figure S7). After the second H_2_ exposure cycle, the IR signal was detected primarily at the rim of the NPs and at sites characterized by a high density of surface defects.

AFM-IR mapping also showed complete SO_*x*_ desorption from the silica substrate. Thus, IR nanospectroscopy measurements reveal the selective removal of SO_*x*_ from the center of the NPs by exposure to H_2_, while the SO_*x*_ coverage essentially remains constant along the rim of the NP, consistent with previously observed site-dependent selectivity.^74,75^

To provide a chemical rationale for the experimental results, *ab initio* calculations were performed within the framework of DFT. The SO_*x*_/Pd and SO_*x*_/PdH systems were modeled by a unit cell consisting of a Pd or a PdH slab with either a lone SO_*x*_ (*x* = 1–4) molecule or an evenly spaced close-packed array of SO_*x*_ molecules. The surface at the center of the nanoparticle was modeled as a flat (111) surface, and the rim of the NP, which is more corrugated than the center, was modeled as a rough surface. PdH was modeled with a Pd:H ratio of 1:0.6875 in accordance with the experiment (Figure S8).^80^ The binding energy of SO_2_ on flat PdH (111) (Figure 6a) is lower by approximately 1.0 eV than on flat Pd(111), indicating a significantly weaker interaction of SO_2_ on PdH(111) in comparison to Pd(111). Similar results were obtained for SO_*x*_ (*x* = 1–4) in which the binding energy values were lower by 1.0–1.5 eV on flat PdH(111) than on flat Pd(111) (Figure 7). This trend rationalizes the probed desorption of SO_*x*_ from the central part of Pd NPs after exposure to H_2_.

**Figure 6:**
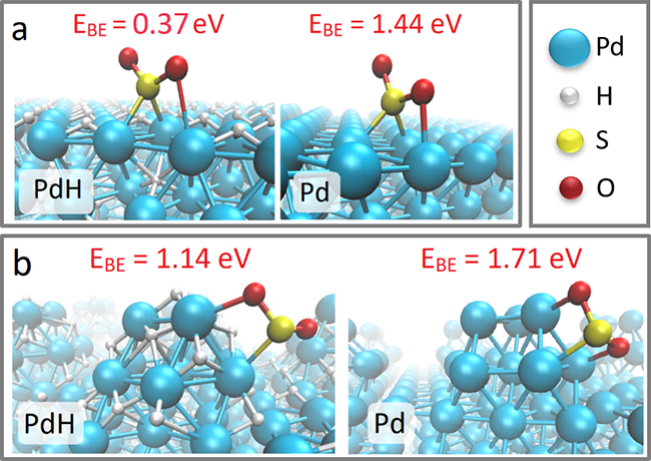
Representative DFT calculations of SO_2_ binding modes (a) on flat PdH(111) and Pd(111) slabs (left and right panels, respectively) and (b) on PdH and Pd islands (left and right panels, respectively). Sulfur atoms are shown in yellow, the O atoms in red, the Pd atoms in cyan, and the H atoms in gray. The binding energy of SO_2_ on various surfaces is marked as *E*_BE_.

**Figure 7:**
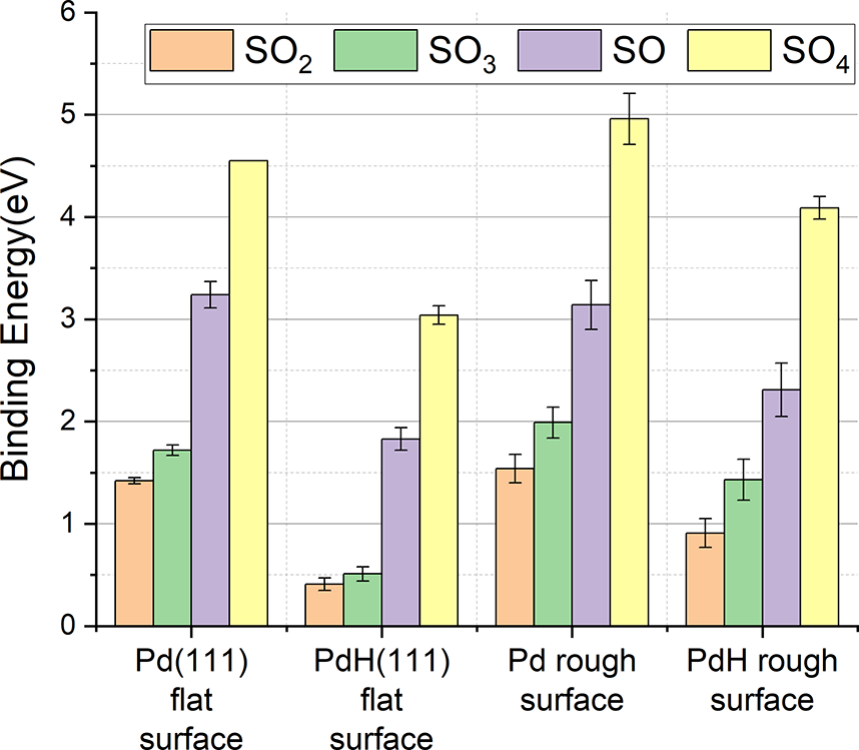
Mean binding energies (eV) of lone SO_*x*_ on different surfaces. Orange columns show the binding energies for SO_2_, green columns show the binding energies for SO_3_, purple columns show the binding energies for SO, and yellow columns show the binding energies for SO_4_. Standard deviations in binding energies due to SO_*x*_ orientation, binding site position on rough surfaces, and local H vacancy distribution near the binding site in PdH are shown by error bars.

Binding energies of SO_*x*_ depended strongly on the binding species, with the order of binding strength as follows: SO_4_ > SO > SO_3_ > SO_2_, as previously reported for Pd(111).^76^ While binding energies of SO_*x*_ on PdH(111) were significantly lower than on Pd(111), the absolute binding energies of SO and SO_4_ are still high, and therefore, they would not be expected to desorb at room temperature. Hence, in light of the experimentally observed desorption of SO_*x*_ following H_2_ exposure, we hypothesize that the main SO_*x*_ species on the nanoparticle surface were SO_2_ and/or SO_3_. It has been demonstrated that SO_2_ and SO_3_ are the main products typically appearing in the early stages of sulfur poisoning with SO_4_ being produced in later stages of oxidation.^43,76–78^ Additionally, it is possible that exposure to hydrogen gas has partially reduced SO_4_ to SO_2_ and SO_3_, while it is known that SO shows higher stability.^79^

Mean binding energies of SO_*x*_ to the different surfaces are reported in Figure 7 for an isolated SO_*x*_ and in Figure S9 for the closely packed arrays of molecules. Variance in binding energies to a specific surface occurs due to different SO_*x*_ binding orientations, different binding sites (on rough surfaces and PdH), and varying local distributions of H vacancies (PdH).

The adsorption pattern on a rough surface, which simulates the morphology at the rim of the NP, was different from the one probed for flat surfaces. The mean binding energy of SO_*x*_ to rough Pd surfaces was generally larger (by up to 0.5 eV) than that of flat Pd(111) surfaces (Figures 6b and 7).^77^ Moreover, higher binding energy values were calculated (by 0.4–1.1 eV) for SO_*x*_ adsorption on rough PdH surfaces compared to that on flat PdH(111). Hence, SO_*x*_ should not detach from the rough rim of the nanoparticle even after exposure to H_2_, in agreement with the experimental findings. These findings provide energetic reasoning for the experimentally observed selective desorption of SO_*x*_ from the central part of the NP following H diffusion into the Pd NP.

In order to compare the single particle AFM measurements of hydrogen (de)­sorption with hydrogen sorption in a large ensemble of nominally identical Pd NPs, we fabricated a 1 cm^2^ quasi-random array of Pd NPs by hole-mask colloidal lithography and studied the impact of SO_*x*_ poisoning on hydrogen (de)­sorption using plasmonic hydrogen sensing measurements (Figure S10). In such measurements, the spectral shift of the localized surface plasmon resonance of the Pd NPs is tracked since it has been demonstrated both theoretically and experimentally to be proportional to the hydrogen concentration in the NPs.^80,81^ Notably, the *t*_90_ response time of the poisoned Pd NPs, which is the time required to reach 90% of the spectral peak shift upon an applied hydrogen concentration change, increased by ∼10-fold in comparison to the nonpoisoned NPs (Figure S11). However, the absolute peak shift obtained in the hydrogenated state was lower by only 15% for the poisoned NPs, in comparison with the pristine ones. Hence, the plasmonic measurements indicate that the overall hydrogen uptake was only weakly influenced by SO_*x*_, while (de)­sorption rates were highly influenced by surface poisoning. This result specifies that the presence of SO_*x*_ on the NPs’ rim lowered the kinetic rate for hydrogen sorption. This can be rationalized mechanistically since SO_*x*_ molecules on Pd NPs block the highly reactive surface sites located at the NP rim and thus lead to slower kinetics, but should not affect the overall sorption affinity.

Single particle analysis showed that exposure of pristine and poisoned NPs to H_2_ led to an average increase of 11.3% and 5.4% in the NP’s volume, respectively (Figure S5). This variance between single particle and ensemble-based measurements indicates that the (de)­sorption kinetics of poisoned NPs probed by AFM was much slower and thus did not reach their equilibrium state under the IR nanospectroscopy measurement conditions. The slower (de)­sorption kinetics in the experimental AFM-IR cell was correlated to a lower relative pressure of H_2_. The impact of poisoning on the kinetics in the AFM-IR cell was further validated by KPFM measurements, as delineated in the following paragraphs.

*Operando* KPFM measurements were performed on the same samples in order to probe the influence of SO_*x*_ poisoning on the electronic properties of Pd NPs as the transformation from metal to metal hydride is expected to impact the work function value. KPFM measurements are sensitive to surface properties and can shed light on the hydrogenation on the particle’s top layers. KPFM measurements probe the contact potential difference (CPD) changes (eq 1), which reflect the difference in work function (φ) between the tip and sample, influenced by surface potential.^82^

1 $$CPD=\frac{1}{e}\left(\phi_{tip}-\phi_{\mathrm{sample}}\right)$$

AFM topography and CPD mapping are shown for pristine (Figure 8a,b–e, respectively) and poisoned (Figure 8f,g–j, respectively) Pd NPs before and after their exposure to alternating cycles of N_2_ and H_2_. CPD measurements show that for both the pristine and poisoned NPs, there is an increase in the signal intensity after exposure to H_2_, which corresponds to a decrease in the work function values of the NPs. This is in good agreement with reported electronic state variation and surface potential,^83,84^ which identified that the adsorbed hydrogen donates electrons to the Pd surface, thereby increasing the electron density and accordingly decreasing the surface dipole and work function.^85–87^ Due to the differences in the measurement mode of AFM versus KPFM, the effective interaction area in KPFM measurements is larger than that in AFM topography measurements, leading to a broader apparent particle size in the CPD maps.^88,89^

**Figure 8:**
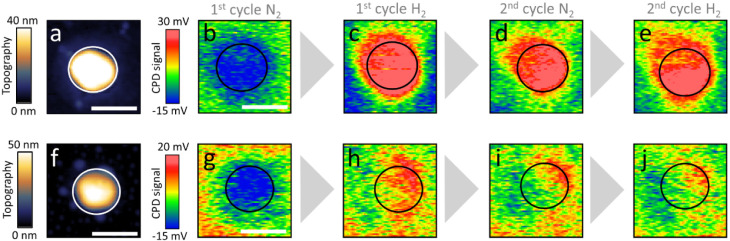
AFM topography of a pristine (a) and poisoned (f) Pd NP. KPFM measurements of the pristine (b–e) and poisoned (g–j) NPs that are shown in (a) and (b), respectively, during consecutive exposure to N_2_ and H_2_. The scale bar represents 200 nm.

CPD mapping of the pristine and poisoned NPs shows changes in CPD values of up to 50 and 25 mV, respectively, upon exposure to H_2_, correlated to a decrease in the work function values of the NPs due to hydrogen sorption (Figure 8c,h, respectively). The difference in the maximum CPD values between the pristine and poisoned NPs is correlated with the smaller hydrogen-induced changes in the volume of the poisoned NPs, in comparison to the pristine NPs. Analysis of the volume changes in the pristine and poisoned Pd NPs following their exposure to H_2_ (shown in Figure 4) reveals atomic percentages of hydrogen of 40% and 28%, respectively. These results further support the large variations in the KPFM signals of the pristine and poisoned Pd NPs.

Variations were also identified in the homogeneity of the signal across the NP. While a similar signal amplitude was identified across the pristine NP, larger variations were detected in the poisoned NP following its exposure to H_2_, attributed to the blocking of active sites by chemisorbed SO_*x*_ species. The nonhomogeneous CPD signal along the NP’s rim reflects the structural variance and heterogeneity in the SO_*x*_ adsorption pattern that can locally impact and change the SO_*x*_ sorption affinity.

After exposure to two cycles of H_2_, both the pristine and poisoned NPs demonstrated intraparticle variations in CPD values. The formation of such heterogeneity was correlated to different nanocrystals that construct the Pd NP. Such heterogeneity was also identified by transmission electron microscopy measurements (Figure S4) and was previously described.^47,49–51,70^ A higher level of heterogeneity was detected on the poisoned Pd NP and attributed to the presence of SO_*x*_ species that lowered the hydrogen sorption kinetic rate on the surface of the NP. The integration of AFM and KPFM measurements therefore complements the impact of hydrogen (de)­sorption on the surface and bulk of the NP.

While significant volume changes were observed during the second cycle of N_2_ and H_2_ exposure, smaller CPD changes were observed for both the poisoned and pristine NP (Figure 8d-e,i-j, respectively, and Figure S12). The KPFM sensitivity to the top layers of the sample, in which hydrogen is strongly bonded, induced smaller changes in the KPFM values after the initial exposure to H_2_. Interestingly, the CPD values of Pd NPs retained their original values after extended exposure (70 h) to N_2_ (Figure S13). Longer N_2_ exposure duration provided sufficient time for hydrogen desorption from the pristine and poisoned NP surface, demonstrating the reversibility of the hydrogen (de)­sorption process from the surface and bulk of Pd NPs. It should be noted that CPD measurements did not show noticeable differences between the CPD values at the rim and the center of the NP, while in the AFM-IR measurements, SO_*x*_ species were mainly probed along the rim of the NP following exposure to two cycles of H_2_ environment. This can be attributed to the lower resolution of KPFM imaging, which makes it challenging to selectively probe the signal that originates from the rim, since this signal can be easily shadowed by the signal from the interior part of the particle.

Integration of the single particle and ensemble-based results shows that SO_*x*_ poisoning induced an overall decrease in H_2_ (de)­sorption kinetics but led to a relatively minor effect on the overall sorption affinity. These results demonstrate that although SO_*x*_ molecules were partially removed from the surface of the particle following consecutive exposure to H_2_, they still have a dominant impact on the (de)­sorption kinetics. SO_*x*_ molecules selectively block active sites on the NPs, thus acting as a “molecular cork” that inhibits the uptake and desorption of surface H atoms.^90,91^ This effect lowered the kinetic rate for (de)­sorption, but, as demonstrated experimentally, did not induce a dominant impact on the hydrogen sorption affinity of the Pd NPs.

## Conclusions

High spatial resolution analysis revealed that sulfur poisoning significantly affects the kinetics of hydrogen (de)­sorption on Pd NPs by selectively poisoning highly reactive surface sites that are mostly located on the rim of the NP. IR nanospectroscopy mapping demonstrated that sulfur species were preferentially anchored to the particles’ rim and poisoned highly reactive surface sites, resulting in a lower hydrogen sorption rate. The selective desorption pattern of SO_*x*_ was rationalized by *ab initio* DFT calculations that showed a large decrease in the binding energy of SO_*x*_ species on the Pd hydride (111) facet compared to that on the metallic Pd (111) facet. However, rough surface sites, such as those located on the particle’s periphery, showed stronger SO_*x*_ binding energies, in particular on rough Pd hydride compared to flat Pd hydride, rationalizing the persistent adsorption of SO_*x*_ on these sites. Ensemble-based plasmonic measurements showed that sulfur poisoning lowered the kinetic rates for hydrogen (de)­sorption on poisoned NPs, correlated to selective blocking of highly reactive surface sites, but led to relatively small changes in the overall hydrogen sorption affinity of poisoned Pd NPs. Contact potential difference measurements showed the correlation between hydrogen sorption affinity and the Pd to PdH_*x*_ phase transition and revealed that the top layers of the NP are characterized by slower (de)­sorption kinetics in comparison to the NP bulk. The integration of single particle and ensemble-based measurements shows that sulfur poisoning deactivated highly reactive surface sites, mostly located at the particle’s rim. The selective poisoning resulted in slower hydrogen (de)­sorption kinetics, demonstrating the impact of trace poisoning species on the reactivity pattern.

## Methods Section

### Nanofabrication of Pd NP Samples

Pd nanodisk arrays were fabricated on 1 × 1 cm fused silica substrates using Hole-mask Colloidal Lithography (HCL).^66^ The particles had a nominal size of 190 nm (diameter) and 25 nm (height), covering approximately 10% of the total sample area. Subsequent annealing was performed at 500 °C for 18 h under a flow of 2 vol % H_2_ in Ar. Statistical analysis of the average size of the particles after annealing is presented in Figure S1.

### Single Particle AFM-IR and KPFM Measurements

Atomic force microscopy infrared (AFM-IR) and Kelvin probe force microscopy (KPFM) measurements were performed in a tapping mode using a nanoIR-3 setup (Anasys, Bruker). The AFM-IR setup is equipped with a Bruker Hyperspectral QCL laser source (790–1950 cm^–1^) and gold-coated Si probes with a nominal diameter of ∼25 nm, resonance frequencies of 75 ± 15 kHz, and spring constants of 1–7N m^–1^. The averaged spectral acquisition time was 5 s per spectrum with a spectral resolution of 2 cm^–1^. IR spectra were collected by averaging five single spectra taken from a single point on the particle. KPFM measurements were performed using Pt-Ir probes with a nominal diameter of ∼25 nm, resonance frequencies of 62 ± 14 kHz, and spring constants of 1–6N m^–1^. Nano-IR measurements were conducted *in situ* at room temperature under exposure to varying gas environments (1 atm of N_2_ or 1 atm of 100:1 N_2_:H_2_ for 60 min), with humidity less than 10%.

### H_2_SO_4_ Poisoning

Sulfur poisoning of the Pd NPs was performed by immersing the sample in 10 Mm H_2_SO_4_ aqueous solution for 10 min at 25 °C. Then, the sample was dried on a hot plate in air at 100–110 °C for 10 min to remove any physisorbed residues.

### XPS Measurements

X-ray photoelectron spectroscopy (XPS) measurements were performed using a Kratos AXIS Supra spectrometer (Kratos Analytical) with an Al Kα monochromatic X-ray source (1486.6 eV). The XPS spectra were acquired with a takeoff angle of 90° (normal to the analyzer), a pass energy of 20 eV, and a step size of 0.1 eV; the vacuum condition in the chamber was 2 × 10^–9^ Torr. The binding energies were calibrated according to the C 1s XPS peak position (B.E. = 285.0 eV). Data were collected and analyzed by using the ESCApe processing program (Kratos Analytical) and Casa XPS.

### FIB-TEM Measurements

Lamella of Pd NPs for STEM imaging was prepared by using a focused ion beam (FIB) in a Helios NanoLab 460F1 setup. Scanning TEM (STEM) images were performed using an aberration probe-corrected Themis Z G3 (Thermo Fisher Scientific) operated at 300 kV, equipped with an annular dark field detector (HAADF) for the diffraction pattern of a single Pd NP.

### Plasmonic Hydrogen Sorption Measurements on Pd Nanoparticle Arrays

The plasmonic measurements were performed in a custom-built reactor chamber that is composed of a customized DN 16 CF spacer flange (Pfeiffer Vacuum), equipped with a gas inlet and outlet, and two fused-silica viewports (1.33 in. CF Flange, Accu-Glass). The effective chamber volume is ca. 1.5 mL. The gas flow rates were controlled by mass flow controllers (EL-Flow Select series, Bronkhorst High-Tech). The purpose of this reactor is to enable kinetics measurements, and therefore, the setup is equipped with 2 units of 3-way valve switches (Peter Paul Electronics Co., Inc.). These switches are connected to the background gas supply and the hydrogen mixture supply. The benefit of having these switches is that the gas mixture can be prepared before reaching the reactor, thus eliminating any gas mixing time factors and uncertainties related to the mass flow controllers.

The sample inside the chamber was illuminated by using an unpolarized halogen white light source (AvaLight-HAL, Avantes) and an optical fiber equipped with a collimating lens. The transmitted light was collected and analyzed by using a fiber-coupled fixed-grating spectrometer (StarLine AvaSpec-ULS2048CL-EVO). The temperature was controlled with a heating coil wrapped around the chamber and a temperature controller (Eurotherm 3216) in a feedback loop manner, where the sample surface temperature inside the chamber was continuously used as an input. Recording of the optical response was performed by using the InsplorionM8 software (Insplorion AB). The optical descriptor for the Pd NP plasmonic resonance used here is the centroid position (center of mass of the plasmonic peak), which is acquired by following a 20th-order polynomial fitting approach.^92^ The plasmonic measurements were conducted *in situ* at *T* = 27 °C under exposure to 99.99% Ar for 60 min, followed by exposure to a mixture of 95% Ar and 5% H_2_ for 60 min. Finally, this protocol was repeated for a total of 2 cycles.

### Density Functional Calculations

All electronic structure calculations were performed within the framework of density functional theory (DFT), using the plane-wave-based Vienna ab initio simulation package (VASP)^93^ with PAW^94,95^ pseudopotentials and the nonlocal correlation functional optPBE-VdW.^96–99^ This functional was chosen because it gives a good prediction of the energy of dissociative adsorption of H_2_ on the Pd(111) surface and also accounts for dispersion interactions.^100^ Results were converged to an accuracy of approximately 0.01 eV, in relation to the cutoff energy for the planewave basis (converged at 400 eV), the vacuum length (set at 12 Å above the metal surface), and the k-point mesh density (a 4 × 4 × 1 Γ-centered mesh).

The Pd(111)/SO_*x*_ complex (*x* = 1–4) was modeled by a unit cell consisting of a Pd slab of 6 layers of 4 × 4 Pd atoms in the horizontal plane and either a lone SO_*x*_ molecule (see, e.g., Figure 6a) or an evenly spaced close-packed array of SO_*x*_ molecules. The structures were geometrically optimized using ionic relaxation with the conjugate gradient algorithm until the convergence criteria of all forces smaller than 5 meV/Å was reached. All atoms were free to move except the bottom two Pd layers, which were kept fixed with the interatomic distance determined by minimizing the energy of bulk Pd. For a unit cell with *n*SO_*x*_ molecules, we calculated the binding energy per unit cell of the SO_*x*_ molecules by comparing the relaxed energy of the SO_*x*_ molecules–Pd complex with the energy of the isolated Pd-relaxed slab plus *n* times the energy of an isolated, relaxed SO_*x*_ molecule placed in a unit cell of the same dimensions as the Pd-slab unit cell. The binding energy per molecule *E*_BE_ was calculated by dividing the binding energy per unit cell by *n* (eq 2). Note that a positive binding energy means the adsorbate is bound to the surface as follows:

2 $$E_{BE}=-\frac{\left[E_{\mathrm{slab}+\mathrm{adsorbed}\;nSO_{x}}-E_{\mathrm{slab}}-nE_{SO_{x}}\right]}{n}$$

Similar calculations were also performed for PdH(111). For bulk PdH, H typically occupies the octahedral crystal positions. However, experiments^80^ show that typically only 0.6–0.7 of these positions are occupied, resulting in PdH with a Pd:H ratio of 0.6–0.7. We therefore removed at random 10 H atoms from a unit supercell of bulk PdH with 32 Pd atoms and 22 H atoms, resulting in a PdH supercell with a Pd:H ratio of 1:0.6875 in accordance with the experiment. Optimization calculations were performed on this cell to obtain the optimal bulk lattice constant and atomic positions. A slab of this optimized PdH was created by placing a vacuum above the (111) surface, fixing the bottom 2 layers of Pd and the bottom layer of H atoms between them at their bulk positions, and allowing the rest of the atoms to relax. This resulted in a slab with H vacancies located randomly throughout the slab. An “annealing” process was then carried out in order to obtain the most stable configuration of H vacancies in the PdH slab: molecular dynamics (MD) simulations were performed on the optimized slab for up to 5 ps at 300, 400, and 500 K, using the canonical ensemble (in which the controlled thermodynamic state variables are the particle number, volume, and temperature (NVT)) with the Nosé-Hoover thermostat.^101,102^ After several picoseconds, the MD simulation was stopped, and the slab was reoptimized. The most stable configuration was chosen and is shown in Figure S8. As can be seen, the H atoms preferentially occupy the surface positions, and the subsurface layer is minimally occupied.

Additional calculations were performed for a rough Pd/PdH surface by creating islands and hollows on the Pd/PdH(111) surface. The rough PdH surface then went through an “annealing” process as described above for the flat PdH surface. During this annealing process, the H atoms in the island interior moved to occupy positions on the island surface, as can be seen in Figure S8.

To model the energetics of SO_*x*_ binding to different Pd and PdH surfaces, we calculated binding energies for lone molecules and closely packed arrays of SO, SO_2_, SO_3_, and SO_4_ to the flat Pd(111) surface, flat PdH(111) surface, rough Pd surface, and rough PdH surface (all PdH calculations were performed on PdH with Pd:H ratios of 1:0.6875, as described above). The binding energies of SO_x_ were calculated in relation to the most stable configuration of H atoms in the substrate obtained via the annealing process described above. For thoroughness, zero-point energy (ZPE) corrections were also calculated for some of the SO_2_ systems and were found to change binding energies by only ±0.04 eV. Note that the zero-point energy corrections were calculated for both SO_2_ bound to the Pd surface and isolated SO_2_ molecules, and the total zero-point energy correction was the difference of these two terms.

It should be noted that binding energies vary with the binding orientation of SO_*x*_ to the surface. Additionally, when binding to the rough surface, the binding energy varies with relation to the specific position of the binding site on the rough surface. Furthermore, when binding to PdH, the binding energy depended on the local configuration of H vacancies close to the binding site. Therefore, we performed binding energy calculations for each SO_*x*_ binding orientation to multiple binding sites on the rough surfaces and on PdH. We found that the minimum energy SO_*x*_ orientation was dependent on the specific binding site for rough surfaces and PdH surfaces. Therefore, when comparing binding energies, we compared the average binding energies for each SO_*x*_ species and surface, as shown in Figures 7 and S9.

Binding energies were calculated for increasingly dense, close-packed arrays of SO_2_ molecules adsorbed on Pd(111). As the arrays became denser, a slight decrease in the binding energy was observed, in agreement with Sharma et al.,^76^ until a large decrease in binding energy occurred when the density was very large (8 SO_2_ molecules per unit cell). Binding energies for close-packed arrays with a specific density (4 SO_*x*_ molecules per unit cell) were also calculated for SO, SO_2_, SO_3_ and SO_4_ on flat and rough Pd/PdH. For SO and SO_2_, there was a slight (0.1 eV or less) reduction in binding energy; for SO_3_, a somewhat larger reduction (∼0.1–0.25 eV); and for SO_4_, there was a more significant (∼0.2–0.4 eV) reduction, as can be seen in Figure S9. The reduction in binding energies for close-packed arrays is not large enough to affect the qualitative conclusions presented in the paper.

## Supplementary Material



## Notes

DFT calculation details, XPS measurements, diameter and height calculations, FIB-TEM cross-sectional analysis, volume analysis, plasmonic and kinetic measurements, and potential surface analysis (PDF)
