# Supplementary material for: Nanoscale Analysis of Sulfur Poisoning Effects on Hydrogen Sorption in Single Pd Nanoparticles
Source: ACS Nano. 2025 Oct 15;19(42):36969–81. doi: 10.1021/acsnano.5c08917 (PMC12574217; doi:10.1021/acsnano.5c08917)
Supplement: Supplementary file 1 [file nn5c08917_si_001.pdf]

Supporting information

Nanoscale Analysis of Sulfur Poisoning Effects on Hydrogen Sorption in Single Pd Nanoparticles

Mazal Kostan-Carmiel[1,2], Athanasios Theodoridis[3], Helen R. Eisenberg[1,4], Tamar Stein[1,4], Christoph Langhammer[3] and Elad Gross[1,2] *

1. Institute of Chemistry, The Hebrew University, Jerusalem 9190401, Israel
2. Center for Nanoscience and Nanotechnology, The Hebrew University, Jerusalem 9190401, Israel
3. Department of Physics, Chalmers University of Technology, Gothenburg SE-412 96, Sweden
4. Fritz Haber Research Center for Molecular Dynamics, The Hebrew University, Jerusalem 9190401, Israel

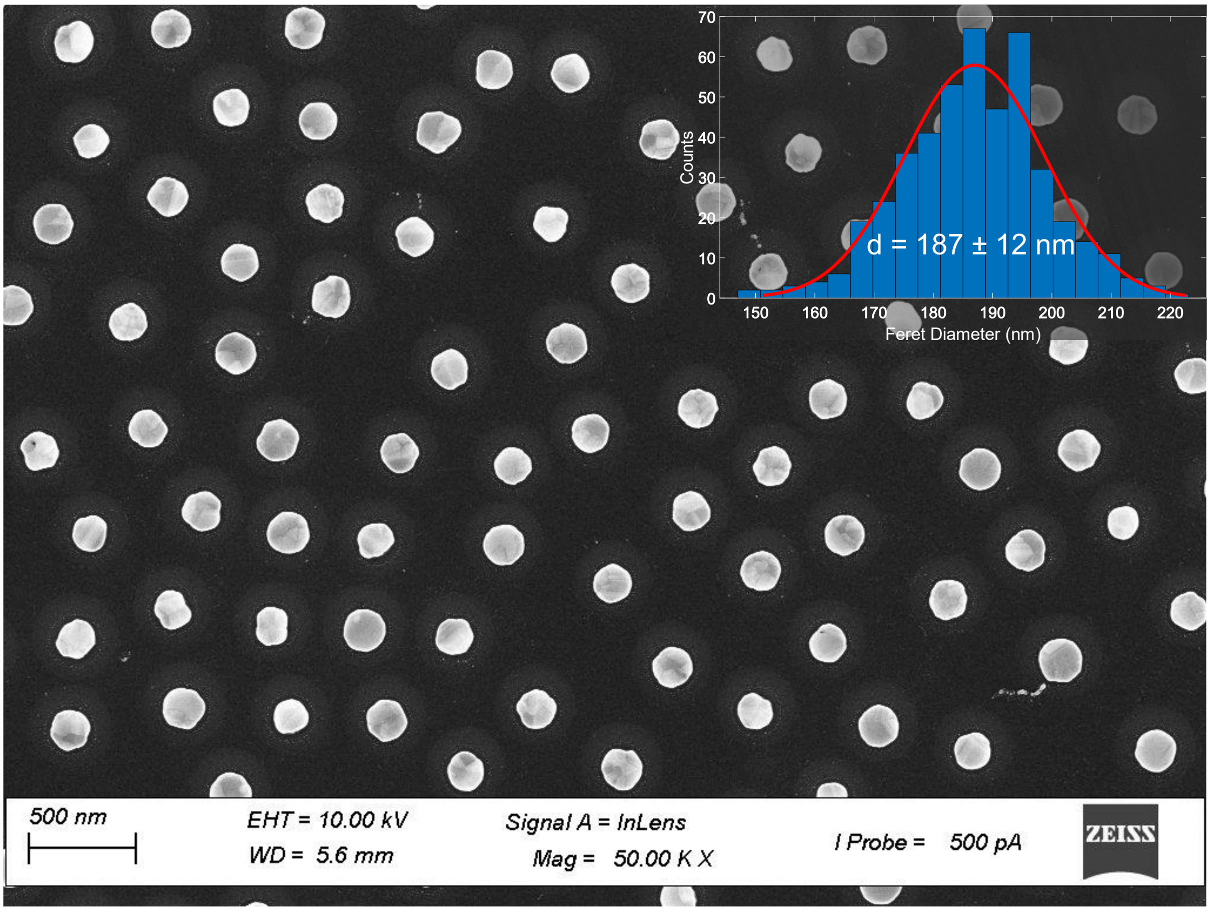

Figure S1. SEM image of Pd NPs. The size distribution (inset) was analyzed by measuring the diameter of ~450 particles from multiple SEM images.

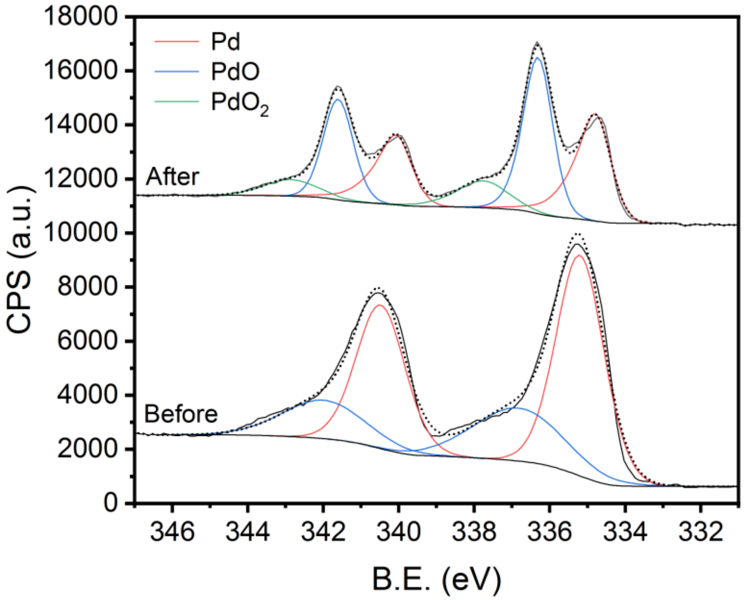

Figure S2. Pd 3d XPS data of Pd NPs before (bottom spectrum) and after (top spectrum) their exposure to H_2SO_4.

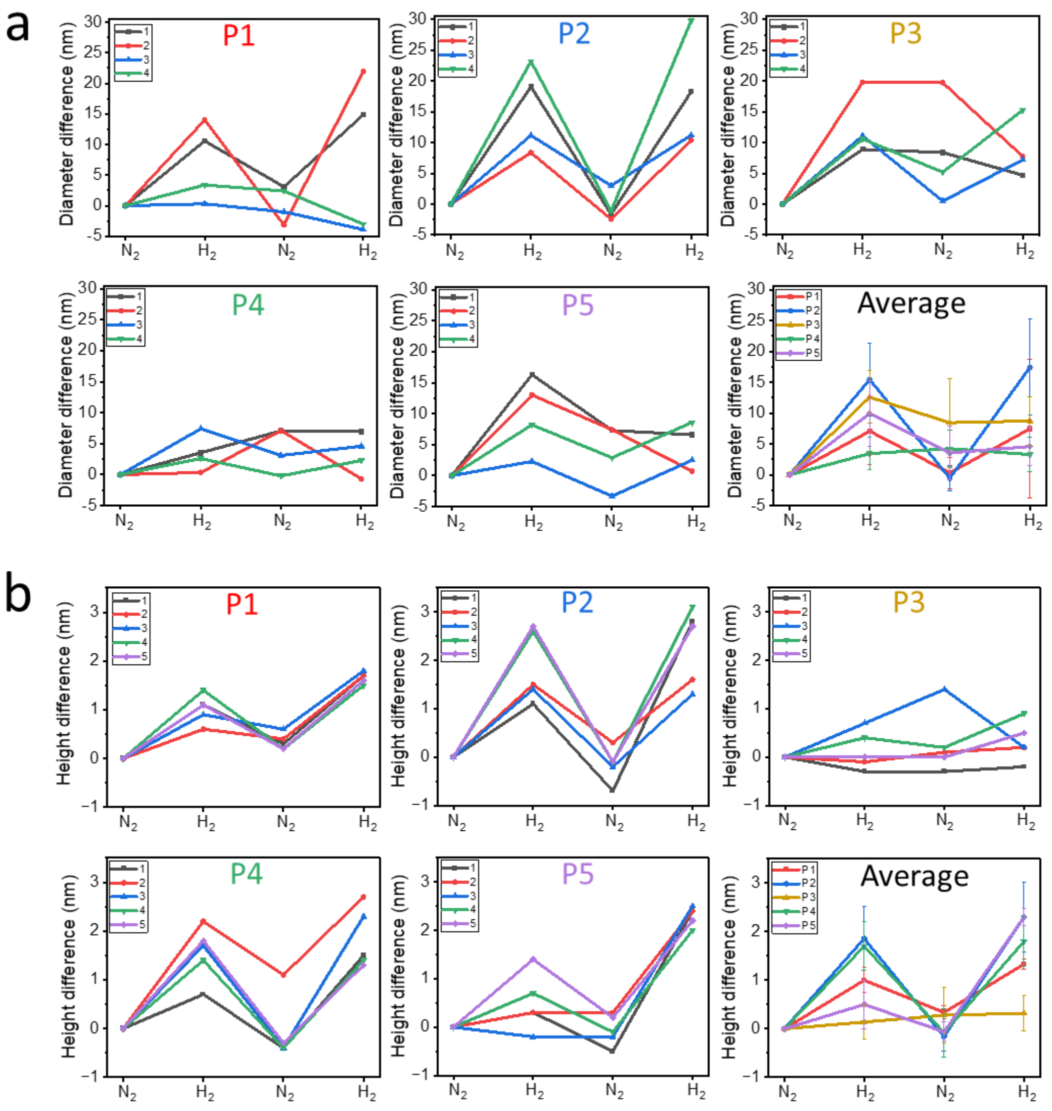

Figure S3. Diameter (a) and height (b) variances of five pristine NPs following consecutive exposure to N_2 and H_2.

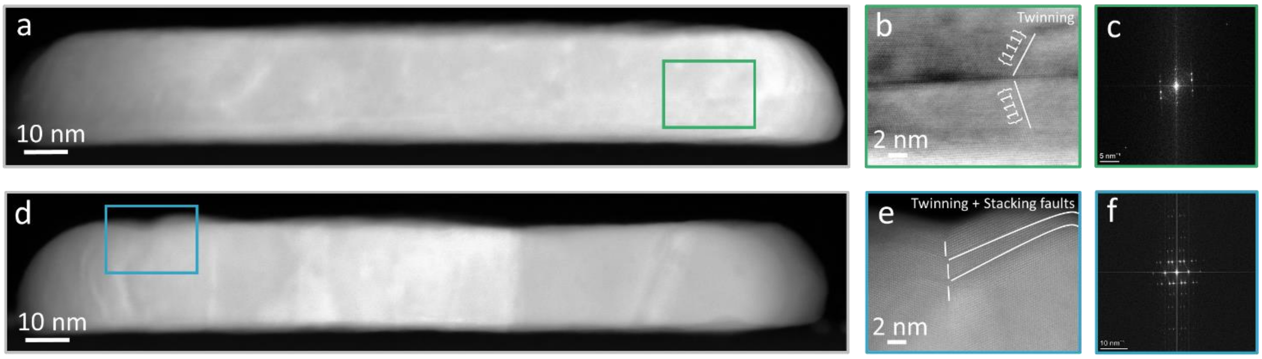

Figure S4. FIB-TEM image of the cross-section of two Pd NPs. (a) A smooth Pd particle exhibited only twinning defects. (b) "Zoom in" on the green-colored area in (a). (c) FFT of the area in (b), revealing a twinned diffraction pattern. (d) A rougher Pd particle with twinning and stacking fault boundaries. (e) "Zoom in" on the blue-colored area in (d). (f) FFT of the area in (e), revealing a twinning and stacking fault diffraction pattern.

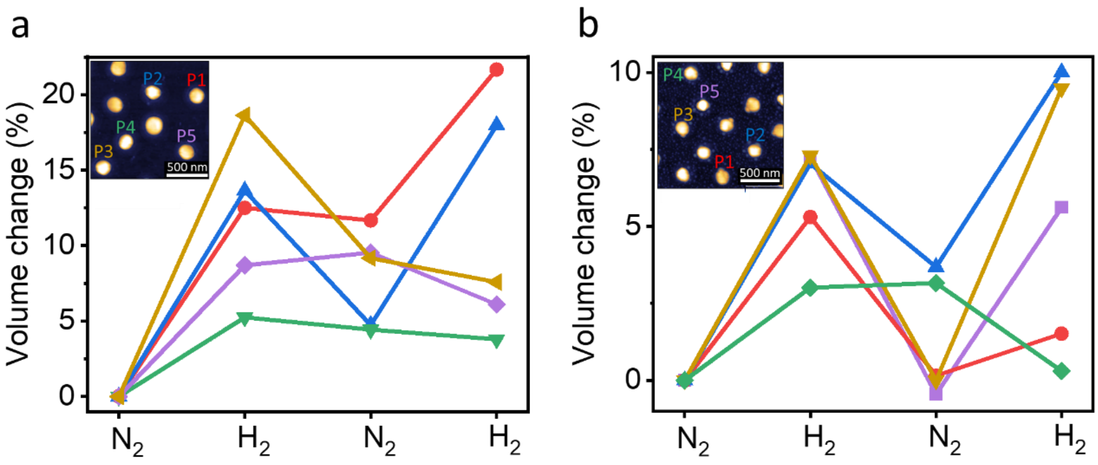

Figure S5. Volume changes of pristine (a) and sulfur-poisoned (b) Pd NPs following alternating exposure to N_2 and H_2.

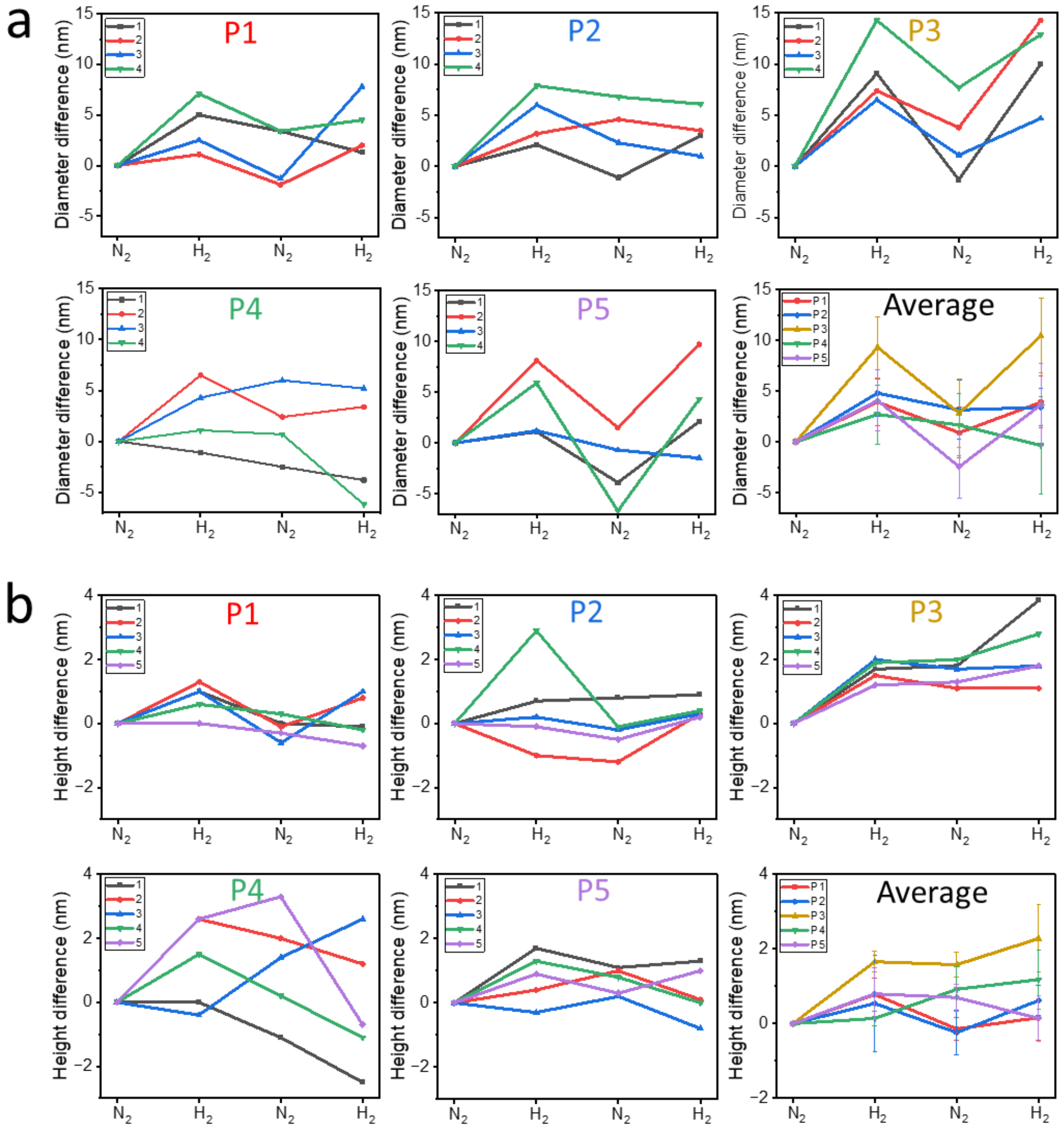

Figure S6. Diameter (a) and height (b) variances of five poisoned NPs following consecutive exposure to N_2 and H_2.

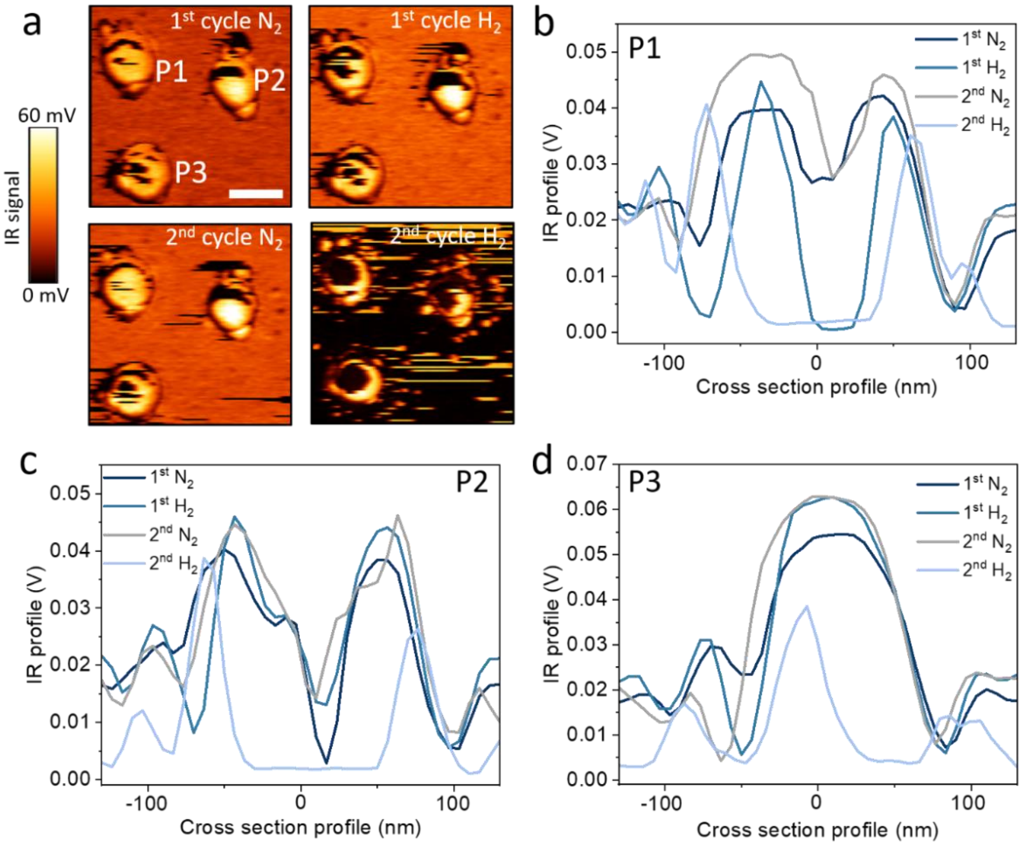

Figure S7. AFM-IR mappings of poisoned Pd NPs were acquired at 1108 cm^{-1} following exposure to N_2 and then H_2 and a second cycle of N_2 and then H_2. (b-d). IR signal profile were acquired for three different NPs following their continuous exposure to exposure to N_2 and H_2. Scale bar represents 200 nm.

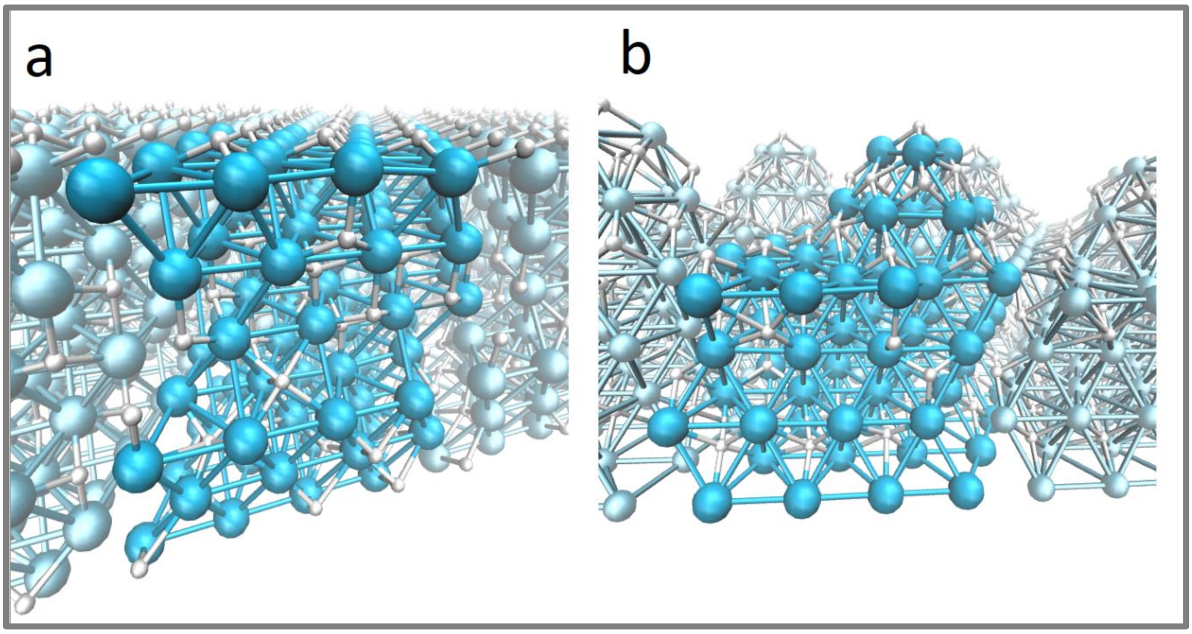

Figure S8. (a) The unit cell for the PdH(111) slab (Pd:H ratio of 0.6875) following annealing and optimization. Pd atoms are shown in cyan, H atoms in grey. Periodically repeated unit cells are shown in faded colors (b) The rough PdH surface (Pd:H ratio of 0.6875) after annealing and optimization.

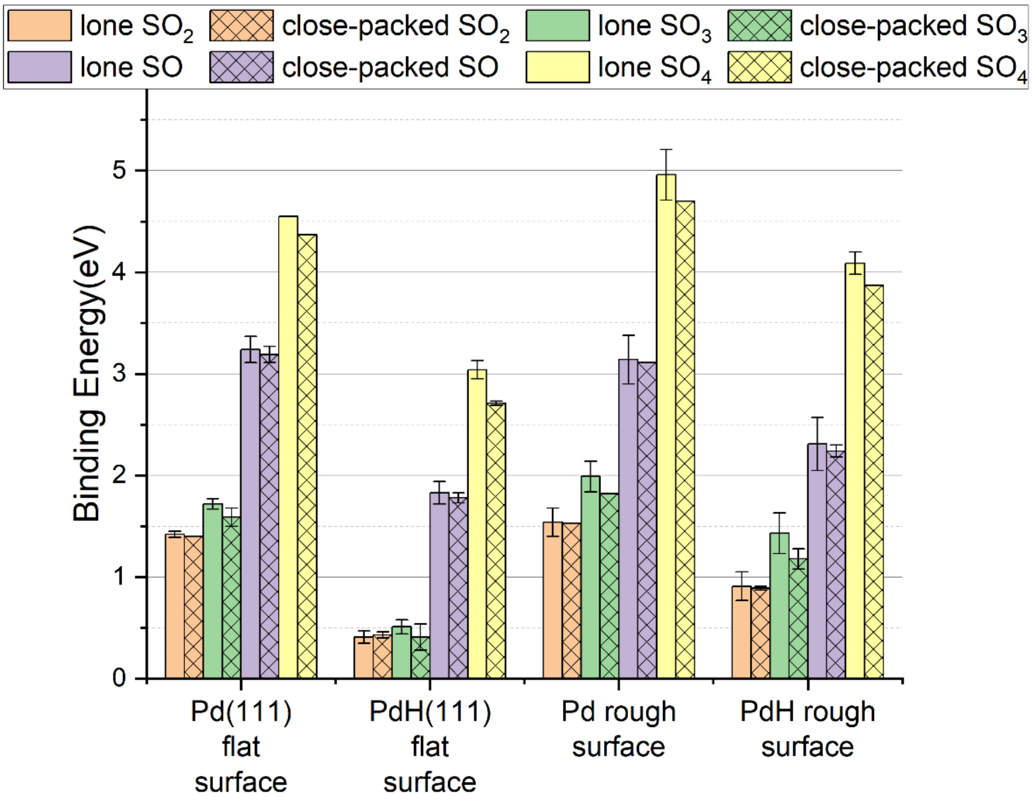

Figure S9. Binding energies for lone SO_x molecules and close-packed arrays of SO_x molecules (4 SO_x molecules per unit cell) on Pd and PdH (Pd:H ratio of 0.6875). The close-packed arrays are shown with a cross-pattern, SO_2 is shown in orange, SO_3 in green, SO in purple and SO_4 in yellow. Standard deviations in binding energies due to SO_x orientation, binding site position on rough surfaces and local H vacancy distribution near the binding site in PdH are shown by error bars.

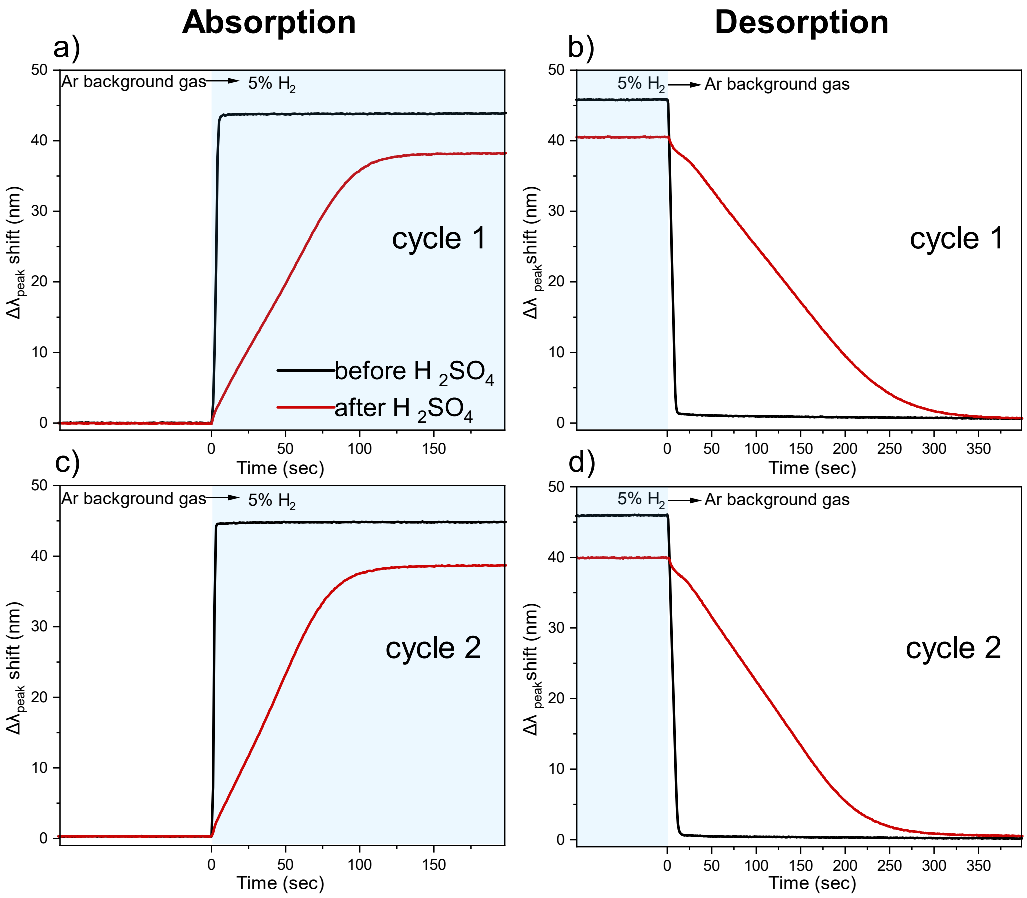

Figure S10. Plasmonic measurements of hydrogen sorption (a,c) and desorption (b,d) kinetics from a pristine and H_2SO_4-poisoned Pd NP array (black and red-colored lines, respectively). The response time for both absorption and desorption is significantly higher after the sample has been poisoned.

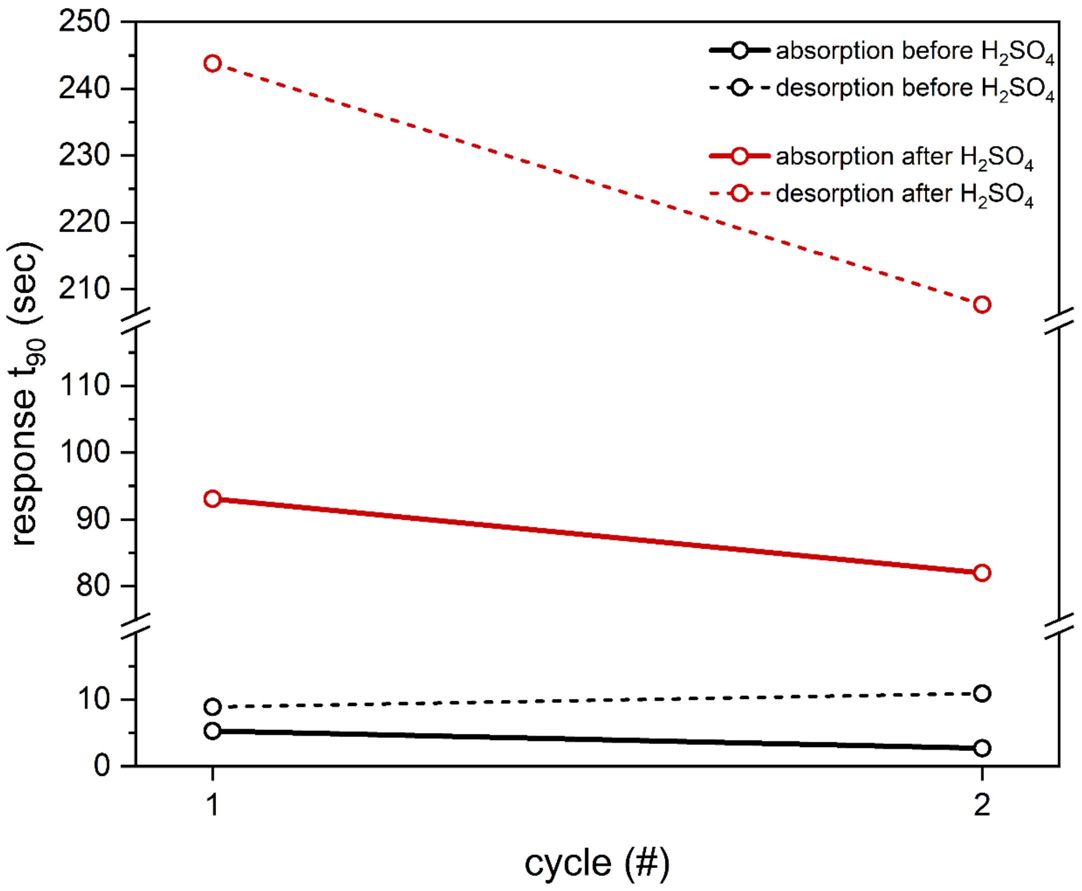

Figure S11. The t_{90} response time of the Pd sensor for the 2 different cycles, extracted from Figure S9. t_{90} corresponds to the time required to reach 90% of the maximum value. The pristine sensor (black) shows a relatively fast response both during sorption and desorption. Upon exposure to H_2SO_4 (red) the response times drastically increase, and the difference between sorption and desorption is more pronounced.

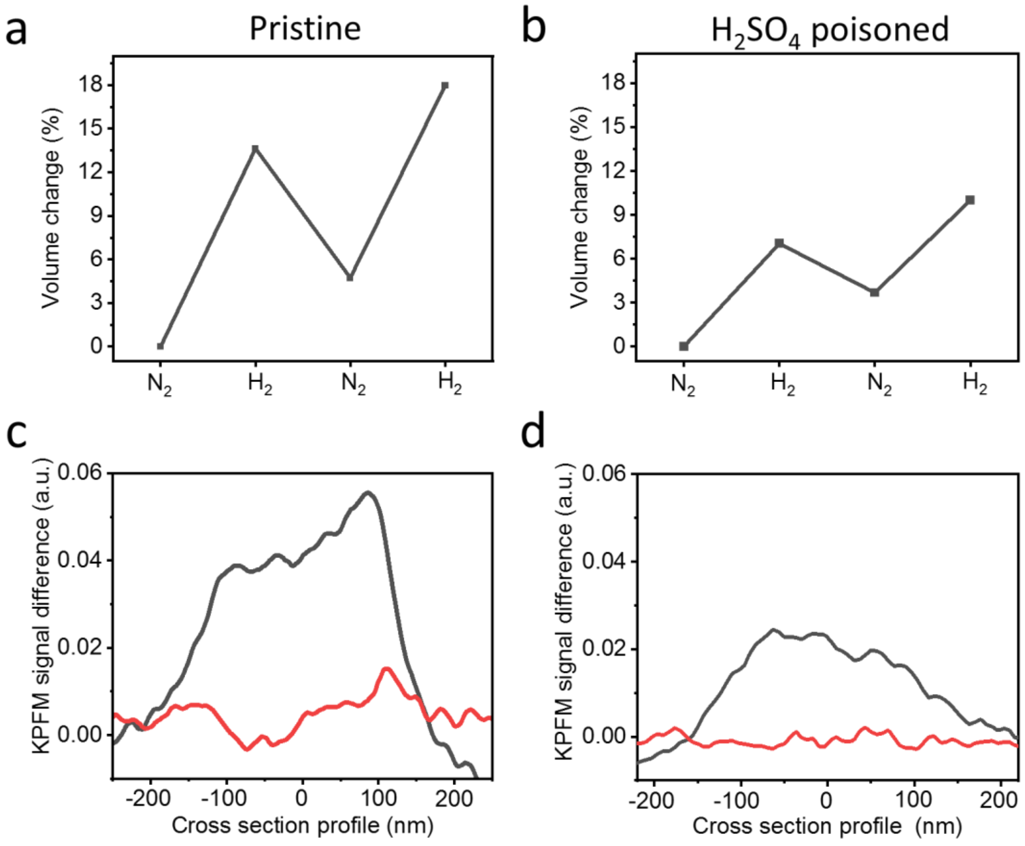

Figure S12. Volume change of a pristine (a) and poisoned (b) Pd NP upon exposure to 1 atm of N_2 and H_2. KPFM signal differences following first and second cycle of exposure to H_2 (black- and red-colored curves, respectively) were measured on the pristine (c) and poisoned (d) Pd NP.

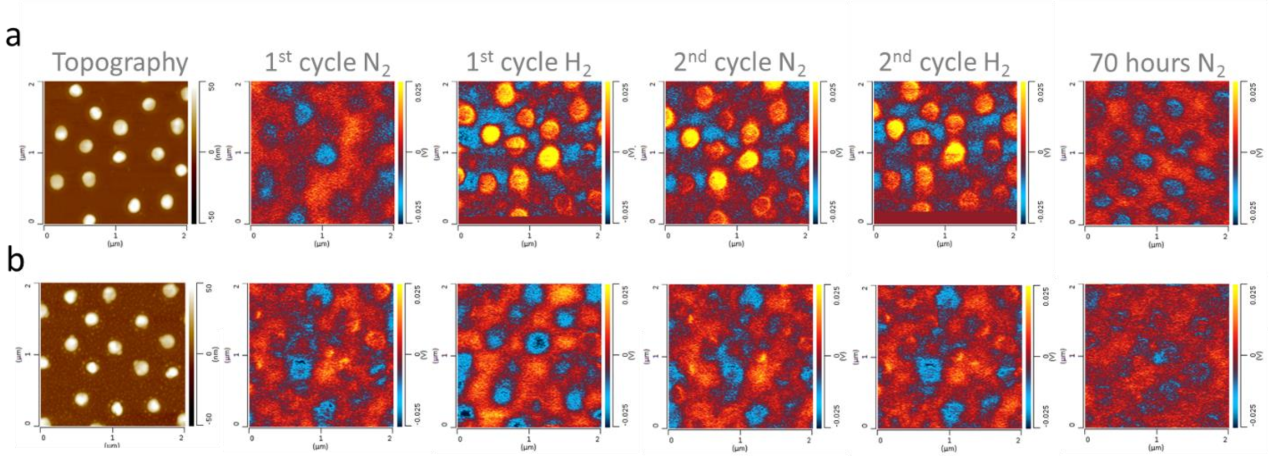

Figure S13. AFM topography image of Pd NPs and their corresponding KPFM mapping following consecutive exposures to N_2 and H_2 before (a) and after (b) exposure to H_2SO_4.
